# Effect of Non-canonical Spatial Symmetry on Subitizing

**DOI:** 10.3389/fpsyg.2021.562762

**Published:** 2021-07-29

**Authors:** Chih-Yen Hsin, Yu-Hui Lo, Philip Tseng

**Affiliations:** ^1^Graduate Institute of Mind, Brain and Consciousness, Taipei Medical University, Taipei City, Taiwan; ^2^Brain and Consciousness Research Center, TMU-Shuang Ho Hospital, Taipei City, Taiwan; ^3^Psychiatric Research Center, Wan Fang Hospital, Taipei Medical University, Taipei City, Taiwan

**Keywords:** subitizing, subitization, enumeration, perceptual grouping, canonical shapes, numerical estimation, groupitizing, pattern recognition

## Abstract

Subitizing refers to ability of people to accurately and effortlessly enumerate a small number of items, with a capacity around four elements. Previous research showed that “canonical” organizations, such as familiar layouts on a dice, can readily improve subitizing performance of people. However, almost all canonical shapes found in the world are also highly symmetrical; therefore, it is unclear whether previously reported facilitative effect of canonical organization is really due to canonicality, or simply driven by spatial symmetry. Here, we investigated the possible effect of symmetry on subitizing by using symmetrical, yet non-canonical, shape structures. These symmetrical layouts were compared with highly controlled random patterns (Experiment 1), as well as fully random and canonical patterns (Experiment 2). Our results showed that symmetry facilitates subitizing performance, but only at set size of 6, suggesting that the effect is insufficient to improve performance of people in the lower or upper range. This was also true, although weaker, in reaction time (RT), error distance measures, and Weber Fractions. On the other hand, canonical layouts produced faster and more accurate subitizing performances across multiple set sizes. We conclude that, although previous findings mixed symmetry in their canonical shapes, their findings on shape canonicality cannot be explained by symmetry alone. We also propose that our symmetrical and canonical results are best explained by the “groupitizing” and pattern recognition accounts, respectively.

## Introduction

Subitizing refers to the ability of the people to rapidly and accurately enumerate a small number of objects. Capacity for subitizing usually falls around three to four elements, and accuracy declines rapidly beyond that (Kaufman et al., 1949; Mandler and Shebo, 1982; Trick and Pylyshyn, 1994). Importantly, studies have shown that the subitizing ability of the preschoolers can be a useful indicator of their arithmetic competence (Gray and Reeve, 2014) and can predict their arithmetic performance in school years after (Hannula-Sormunen et al., 2015).

The association between subitizing and one’s mathematical abilities on surface seems to imply subitizing is numerical in nature. However, recent studies have also shown that subitizing ability does not necessarily predict one’s math abilities (Anobile et al., 2019), as well as that developmental dyscalculia is not always associated with impairment in subitizing ability (Decarli et al., 2020). As such, theorists have proposed other attentional or pattern recognition mechanisms that are not necessarily numerical in nature to account for findings in subitizing. For example, in the case of an attentional account (e.g., Apthorp and Bell, 2015), it has been shown that the capacity of three to four items is not specific to subitizing but has also been found in visual working memory (e.g., Luck and Vogel, 1997), visual attention and selection (e.g., Cowan, 2001; Xu and Chun, 2009), and multiple-object tracking (e.g., Pylyshyn and Storm, 1988; Yantis, 1992). Importantly, subitizing is sensitive to perceptual load manipulation in an attentional blink paradigm (Burr et al., 2010); visual tracking of other objects also reduces subitizing capacity (Chesney and Haladjian, 2011), and even the presence of other-colored shapes can impair subitizing (Liu et al., 2020). These findings seem to converge and confirm a critical role for attention in subitizing performance, and, as such, recent studies have mostly converged toward a common-resource framework (Melcher and Piazza, 2011; Piazza et al., 2011; Cutini and Bonato, 2012; Formoso et al., 2017; but see Shimomura and Kumada, 2011), which has been implicated to involve the parietal cortex (Walsh, 2003; Knops et al., 2014; Pagano et al., 2014; Apthorp and Bell, 2015; Harvey et al., 2015; Bloechle et al., 2018).

In the case of a pattern recognition system, studies have also shown that subitizing performance can benefit from familiar patterns in a visuospatial layout of the dots. For example, Mandler and Shebo (1982) proposed a pattern recognition mechanism where dots are mentally connected to create a visual pattern. Similarly, many studies have also found that “canonically” organized dots (such as patterns on a dice) can also facilitate the reaction time (RT) of the people and accuracy in a subitizing task (Wender and Rothkegel, 2000; Piazza et al., 2002; Krajcsi et al., 2013; Bloechle et al., 2018). Furthermore, even without familiarity, some kind of grouping characteristics, or ensemble statistics, can also improve subitizing performance, suggesting such a pattern recognition process is not purely mnemonic but also perceptual. Indeed, spatially separated clusters, or ensembles, are treated as one individual object (Eisinger et al., 2012), thereby facilitating behavioral performance. Similar facilitation from grouping can also be observed in haptics (Overvliet and Plaisier, 2016). This facilitation from spatial arrangement, even when set size is controlled for, possibly suggests facilitation in visual rapid categorization, which is consistent with suggestion of Trick and Pylyshyn (1994) that subitizing starts as early as the preattentive stage. This perceptual and preattentive process is assumed to occur during the scene categorization or the “gist” stage that can take place in less than 100 ms (Sampanes et al., 2008; Greene and Oliva, 2009) and can further aid the attentional selection process mentioned above. On top of that, familiar spatial arrangement likely further enjoys benefits of rapid categorization from long-term memory, which may be akin to the idea of a schema in other literature domains, where expert chess players can reconstruct a complex chessboard scenario with only a brief exposure.

### Canonicality vs. Symmetry

**FIGURE 1:**
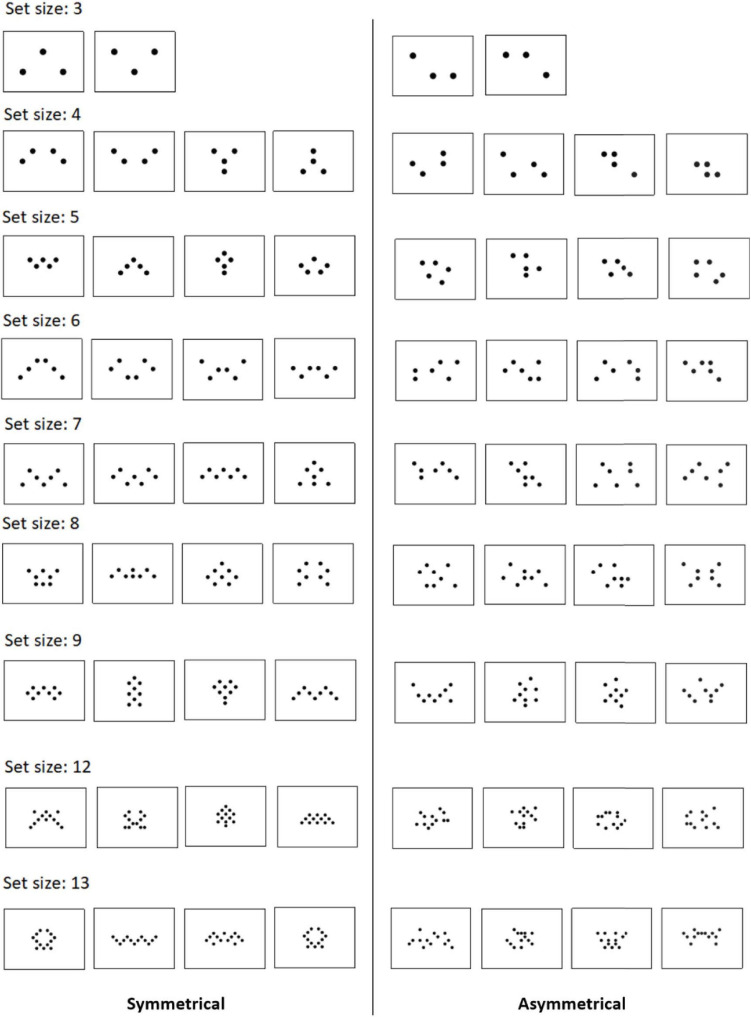
Symmetrical and asymmetrical designs from this study. A number of dots are balanced between left and right sides. Asymmetric versions are created by randomly moving 50% of the dots from their symmetrical counterparts. Odd-numbered set sizes were scaled down by 1 disc. In addition, the black-to-white ratio was kept the same across all set sizes such that the number of black pixels is always the same in every trial throughout the entire experiment regardless of set size.

**FIGURE 2:**
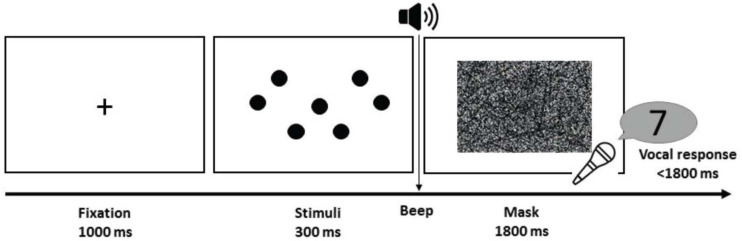
Procedures of the subitizing task. Each trial started with a fixation for 1,000 ms, followed by the stimuli for 300 ms and a visual mask for 1,800 ms. The mask began with a beep, which signaled the participants to verbally respond with a number.

To further explore the effect of perceptual grouping on subitizing, the present study aims to specifically investigate the possible contribution of spatial symmetry. This is related to the literature on pattern recognition and shape canonicality reviewed above. Specifically, those of Wender and Rothkegel (2000); Piazza et al. (2002), Krajcsi et al. (2013), and Bloechle et al. (2018) have all found faster RT or larger enumeration capacity when canonical organizations were used, as opposed to random layouts. However, upon a closer look at the stimuli that were used (Wender and Rothkegel, 2000: page 92, Appendix; Piazza et al., 2002: page 438, Figure 1; Krajcsi et al., 2013: page 230, Figure 2; Bloechle et al., 2018: page 18, Appendix A), it is worth noting that all previous studies blended spatial symmetry in their canonical design, thereby combining the two factors together (Wender and Rothkegel, 2000; Piazza et al., 2002; Krajcsi et al., 2013; Bloechle et al., 2018), and leaving the effect of spatial asymmetry (independent from canonicality) is still an open question.

Indeed, to the best of our knowledge, all canonical designs from previous studies so far also happened to be symmetrical. The only exceptions (i.e., canonical shapes that are *not* symmetrical) from these studies are canonical designs at set sizes 2 and 3, where the dots are often diagonally placed (i.e., a dice layout) or forming a right triangle. However, set sizes 2 and 3 are well within the capacity of the people for subitizing, and thus a ceiling effect is always reported in this range regardless of the spatial layout. One closest manipulation in the literature so far is jittering manipulation of Krajcsi et al. (2013), where they jittered the location of each dot of a canonical shape within a specified (but non-overlapping) grid around each dot. Although this was not the research question of the study, their small- and large-jitter manipulations did maintain shape symmetry, which would partially yield the effect of symmetry (but non-canonical) on subitizing when compared against their total-random condition. However, because these authors maintained spatial separation by using non-overlapping grids around each dot, the canonical structure remained pretty much intact in both jittered conditions (Krajcsi et al., 2013: page 230, Figure 2), which was reflected in their data as well (e.g., no significance difference in subitizing performance between small jitter and canonical condition). Therefore, to the best of our knowledge, there is no study that has examined the effect of spatial symmetry on subitizing without using canonical shapes. This is an important manipulation because (1) the effect of spatial symmetry may have been overshadowed by the effect of shape canonicality (if symmetry is a smaller effect) in previous studies, or alternatively, (2) it is even possible that the effect of shape canonicality from previous studies was mostly driven by spatial symmetry.

To this end, the present study aims to only examine the possible effect of spatial symmetry on subitizing by using symmetrical, yet non-canonical, shape structures. It is important to note that, theoretically, the two factors of symmetry and canonicality can be teased apart by examining either factor alone while controlling for the other. But, realistically, while it is possible to have symmetrical shapes that are non-canonical, there are, virtually, no canonical shapes that are asymmetrical. As such, it is more fruitful to investigate the effect of symmetry by controlling for canonicality, and our results here will also shed light on the results of previous studies.

## Experiment 1

To independently investigate the possible effect of spatial symmetry on subitizing, we created a pool of symmetrical figures that are not canonical. Our rationale is that, if we find comparable effect sizes to previous canonical studies by using symmetry alone, then it is possible that previous findings on canonical shapes may have been driven by the mixed use of spatial symmetry. On the other hand, if we observe a much weaker effect, or even no effect, with symmetry alone in the present study, then it would imply that findings from previous studies, using canonical shapes, were, indeed, driven by canonicality instead of spatial symmetry.

### Methods

#### Participants

Thirty participants (11 males, 19 females, age 20–37, mean age = 21.17) took part in this experiment. All the participants had normal or corrected-to-normal vision. All gave written informed consent prior to their participation and received financial compensation for their time. Data of two participants were excluded from analysis because their accuracy in set size 3 or 4 was lower than 100%. Twenty-eight participants were included in the following analysis. All experimental procedures were approved by the Joint Institutional Review Board of Taipei Medical University, Taiwan.

#### Subitizing Task

The participants sat in a dimly lit room with their chins on a chin rest to perform the experiment. The experiment was performed, using E-Prime 2 Professional Software (Psychology Software Tools). The microphone (for verbal response) and the computer display were placed 5 and 57 cm in front of the chin rest, respectively. In this task, the participants had to subitize dots and to respond vocally in their native language Mandarin. Vocal response latencies were measured, using a voice key, connected with the computer *via* a PST Serial Response Box. The dots were shown at nine set sizes (3, 4, 5, 6, 7, 8, 9, 12, and 13). Set size 3 had two symmetrical and two asymmetrical designs, and other set sizes had four symmetrical and four symmetrical designs, resulting in 34 symmetrical trials and 34 asymmetrical trials. All stimuli were presented in a randomized order. The participants also performed eight practice trials, resulting in a total of 76 trials that took approximately 15 min to complete.

Each symmetrical pattern has its own asymmetrical control, which is created by moving 50% of the dots from the symmetrical pattern (Figure 1). This was to ensure that the asymmetrical versions were not totally random but were created from a symmetrical template, and all set sizes had similar asymmetrical complexity (50% of the dots). Odd-numbered set sizes were scaled down by 1 disc. Importantly, even in asymmetrical trials, the number of dots between the left and right sides was kept the same, such that the symmetrical and asymmetrical trials all had the same amount of dots on both sides, and only differed in terms of symmetrical structure. This was done to avoid the asymmetrical trials being overly heavy on one side, which would introduce potential confounding factors in the responses of the participants.

Furthermore, to control for the amount of black pixels (dots) on the white background, the black-to-white ratio was kept the same across all set sizes. That is, the dots in a three-dot trial would be slightly bigger than a four-dot trial, and much bigger than a 13-dot trial, such that the number of black pixels is always the same in every trial throughout the entire experiment. This is to ensure that the set sizes only differed in numerosity and not overall physical color. Additionally, this design can also help prevent participants from estimating the overall black area without actually subitizing. Note that, however, this design also implies that numerosities and dot size would be inversely correlated. But not doing so might risk the participants not paying much attention to the stimulus and simply estimate the overall black area by squinting their eyes or using peripheral vision (by looking aside). Therefore, the present study opted for controlling for the overall area and requires the participants to focus on the display.

Each trial started with a 1,000-ms fixation cross, followed by 300 ms of dots and a 1,800-ms mask. At the onset of the mask, the participants would hear a beep tone and were to respond vocally with a number within 1,800 ms (Figure 2). Vocal responses were chosen here for two reasons: first, we can get numerical estimates from the participants instead of a binary same/different response. In incorrect trials, this measure becomes useful as it allowed us to compute the *error distance* of this trial (i.e., how wrong the participant was). Second, for practical purposes, verbalizing the numbers is a much more automatic process, and thus eliminating the need for the participants to type their estimates on the keypad, which would inevitably increase the RT of the participants due to unfamiliar response motor mapping. The vocal RT of the participants was derived from the beep offset and the moment of the utterance of the participants (trials that started with “um,” “uh,” or any types of hesitation or sounds that did not correspond to a number were excluded from analysis). Accuracy of each trial was manually inputted by the experimenter by listening to all recorded responses after the experimental session.

#### Secondary Tasks

To gauge whether the subitizing performance of the participants might be related to their mathematical or perceptual abilities, the participants performed two secondary tasks: one arithmetic and one visual, after they had completed the subitizing task. These two tasks were adapted from the working memory task by Foster et al. (2015) but, with the working memory component, removed to only gauge arithmetic and visuospatial abilities of the participants.

**FIGURE 3:**
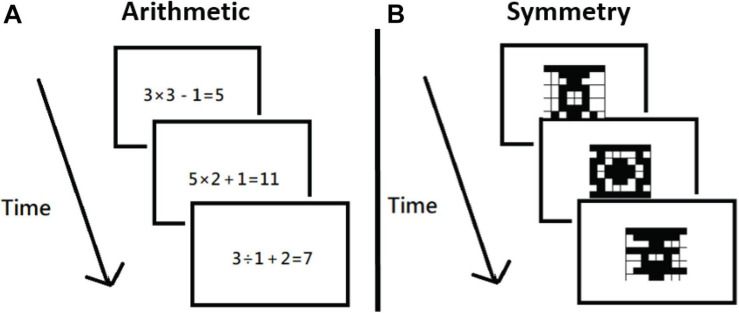
Procedures of the secondary tasks. The participants had to judge correct vs. incorrect within 3,500 ms for the arithmetic task **(A)**, and judge symmetrical vs. asymmetrical within 1,000 ms in the symmetry task **(B)**. These tasks were modified based on what was a working memory task by Foster et al. (2015).

The arithmetic task showed the participants a mathematical equation (e.g., 3 × 3 – 1 = 5) for 3,500 ms, and, within that time frame, the participants had to judge whether the formula was correct or incorrect by pressing C or M, respectively (Figure 3A). In the visual symmetry task, the participants saw an 8 × 8 grid with some spaces filled for 10,000 ms, during which they had to judge whether the filled spaces would form a symmetrical or asymmetrical pattern by pressing C or M, respectively (Figure 3B). Both RT and accuracy data were collected.

#### Statistical Analyses

All accuracy and RT data were analyzed with a repeated-measures two-way ANOVA with the factors of symmetry (symmetrical vs. asymmetrical) and set size (3, 4, 5, 6, 7, 8, 9, 12, and 13). Where applicable, *post hoc* comparisons were done using Bonferroni correction.

Furthermore, because ANOVA may not be the best analysis for accuracy data due to their binomial nature, we also analyzed accuracy data with generalized linear mixed models (GLMM) to test our alternative hypotheses without assumptions about the underlying distribution (Lo and Andrews, 2015)^1^, and we used linear mixed models (LMEM) for RT and error distance data. Symmetrical structure and set size were modeled as fixed effects in the models. For all analyses, ID of the participants was modeled as a random factor. All analyses were conducted using the JASP (version 0.13.1.0) software.

### Results

#### Accuracy

**FIGURE 4:**
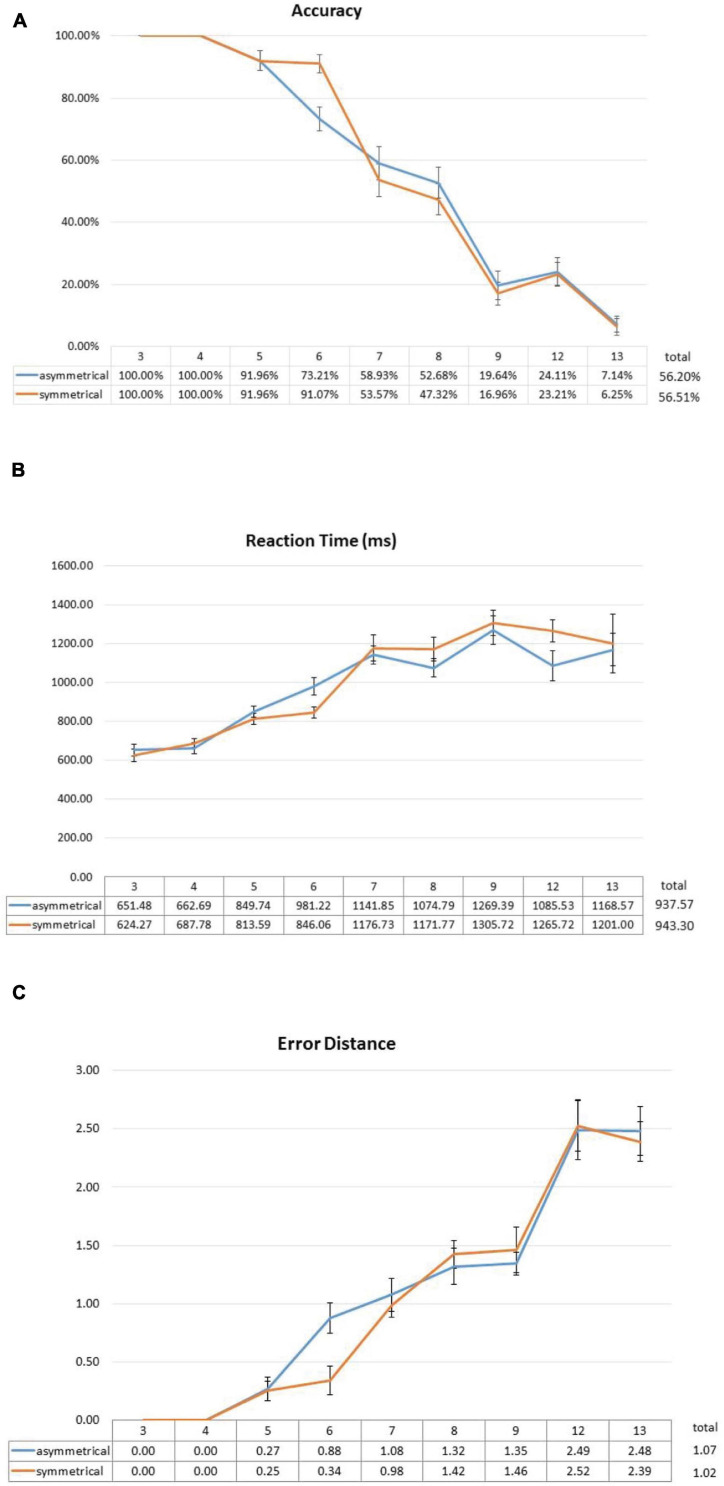
Effect of symmetrical structure on subitizing accuracy **(A)**, RT **(B)**, and error distance **(C)**. The significant interaction in accuracy was mainly driven by the 20% difference at set size 6. Similar effects were also observed in RT and error distance, albeit in much weaker forms. Error bars represent standard error of the mean.

Overall, the participants had 56.36% correct trials, 3.36% no-response trials, and 40.28% incorrect trials. Two-way repeated-measures ANOVA showed no main effect of symmetry [*F*(1,27) < 0.001, *p* = 1], a significant main effect of set size [*F*(8,216) = 164.800, *p* < 0.001], and a significant interaction between symmetry and set size [*F*(8,216) = 2.749, *p* = 0.022 (Greenhouse–Geisser corrected)]. *Post hoc* analyses showed that the interaction was driven by the significant beneficial effect of symmetry at, and only at, set size 6 [*t*(27) = -3.731, *p* = 0.008 (Bonferroni correction)] (Figure 4A) and not at any other set sizes.

We also analyzed the accuracy data of the participants, using a generalized linear mixed-effect model with a logistic link function and a binary probability distribution for modeling the single trial accuracy (i.e., correct or incorrect response). Fixed effects were symmetry, set size and interaction between the two factors. Participant IDs were selected as random factors. We found that symmetry and its interaction with set size did not affect measures of accuracy (symmetry: β = -0.253, *t* = -1.160, *p* = 0.246; interaction: β = 0.033, *t* = 1.209, *p* = 0.227). Only set size significantly affected the accuracy of the participants (β = -0.616, *t* = -22.217, *p* < 0.001).

#### Reaction Time

In terms of RT, of all the correct trials, two-way ANOVA also showed no main effect of symmetry [*F*(1,21) < 0.001, *p* = 0.995] and a significant main effect of set size [*F*(5,105) = 40.731, *p* < 0.001]. Similar to the accuracy results, there was a marginally significant interaction between symmetry and set size [*F*(5,105) = 22.600, *p* = 0.066 (Greenhouse–Geisser corrected)]. From Figure 4B, it can be seen that RTs in the symmetrical condition is also faster, which is similar to the observations from accuracy data.

We also used the linear mixed-effect model for analyzing RT data from correct trials. Fixed effects were symmetry, set size, and interaction between the two factors. Participant IDs were selected as random factors. The interaction among symmetry and set size was significant (β = -8.057, *t* = -2.180, *p* = 0.029). The effects of set size and symmetry also significantly predicted the reaction time of the participants (set size: β = 78.693, *t* = 21.198, *p* < 0.001; symmetry: β = 51.948, *t* = 2.220, *p* = 0.027). The positive coefficient of symmetry in GLMM of RTs indicates significantly faster reaction times during symmetrical structure than asymmetrical one. The predicted reaction time of the participants was equal to 436.843 + 78.693 ^∗^ set size + 51.948 ^∗^ symmetry -8.057 ^∗^ set size ^∗^ symmetry.

#### Error Distance

To further explore the effect of symmetry, we analyzed the 40.28% incorrect trials and calculated “error distance” of each trial. That is, when a participant gets a trial wrong, are they close to getting it correct? (e.g., subitizing 6 as 7) Or are they simply way off (e.g., calling 6 as 9). The rationale is that, if symmetry can really help, it should not only increase the number of correct trials, but it should also push the answers of the people closer to the correct answers even when they get them wrong. To this end, we calculated the error distance for every incorrect trial and, again, conducted a two-way ANOVA with the factors of symmetry and set size. The two-way ANOVA revealed no main effect of symmetry [*F*(1,24) = 0.214, *p* = 0.648], but a significant effect of set size [*F*(8,192) = 75.632, *p* < 0.001], and no significant interaction between the two [*F*(8,192) = 1.249, *p* = 0.294 (Greenhouse–Geisser corrected)]. If we only look at set size 6 (Figure 4C), it also shows the same facilitating effect of symmetry in error distance [*t*(27) = 4.104, *p* = 0.003 (Bonferroni correction)], which is consistent with what we have observed so far in accuracy and RT, although it was not enough to drive the entire ANOVA toward statistical significance.

Overall, we observed a significant interaction between symmetry and set size in accuracy, which is due to the facilitative effect of symmetry at set size 6. This suggests that symmetry does improve the subitizing ability of the people at six items, and such facilitative effect disappears at set size 7 (Figure 4A). This trend of facilitative effect from spatial symmetry is also present in RT and error distance, although the significance level of the two-way interaction gets progressively weaker. The fact that symmetry fails to improve performance of people beyond set size 6 suggests that symmetry is a much weaker effect than canonicality from previous studies; we discuss this in more detail in General Discussion. Using LMEM (with symmetry, set size, and interaction between them as fixed effects, and participant IDs as random factors; only set size showed statistical significance (β = 0.214, *t* = 12.626, *p* < 0.001).

#### Weber Fraction

**FIGURE 5:**
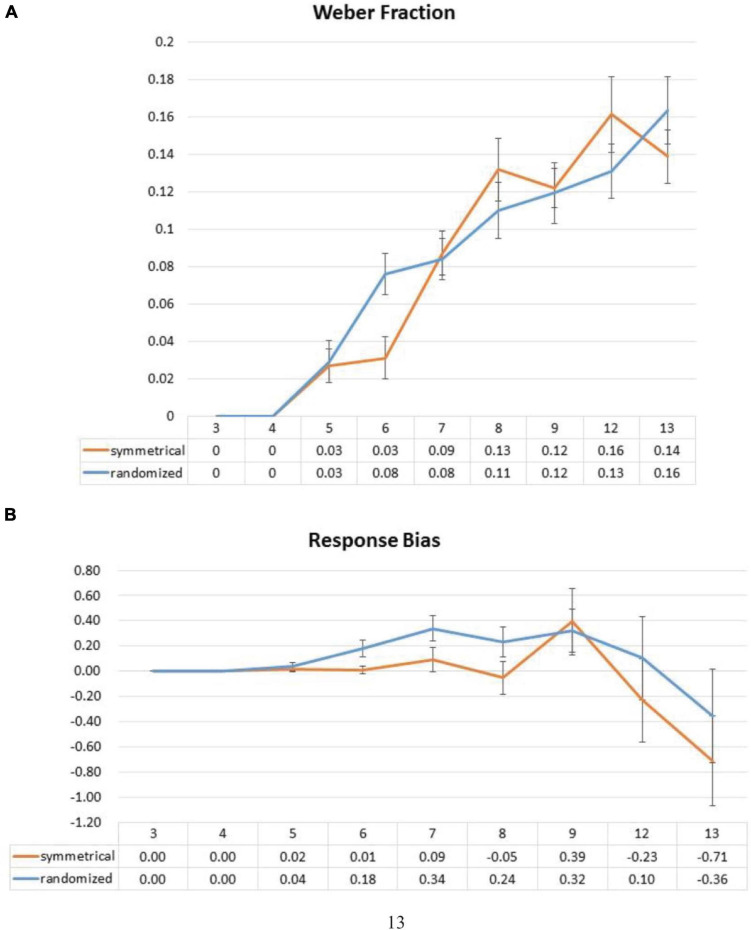
Effect of symmetrical structure on Weber fractions **(A)** and response bias **(B)**. Weber fraction is a ratio of average error in relation to target size by dividing standard deviation of one by perceived numerosity. Response bias is computed by subtracting set size from the average response such that a positive difference would denote overestimation, and vice versa. Error bars represent standard error of the mean.

Previous studies mostly used the Weber fraction to assess response sensory precision of the participants in numerical estimation (Whalen et al., 1999). This is done by dividing standard deviation of one for a particular set size by the corresponding perceived/reported set size to create a ratio of average error in relation to target size. This is because response variability of people tends to increase with the target set size, and the use of this method would not need to exclude correct responses as we have done in the error distance analysis. Here, we submitted these ratios to a two-way ANOVA with factors of symmetry and set size. We did not observe a significant main effect of symmetry [a symmetrical layout: *F*(1,25) = 0.002, *p* = 0.961], but a marginally significant interaction between symmetry and set size [*F*(8,200) = 1.851, *p* = 0.070] (Figure 5A). *Post hoc* analyses showed that the marginally significant interaction was driven by the significant beneficial effect of symmetry at set size 6 [*t*(27) = -3.761, *p* = 0.007 (Bonferroni correction)]. No significant differences were observed at any other set sizes.

#### Response Bias

Because symmetrical structure has been shown to induce an underestimation of the number of components (Apthorp and Bell, 2015) and even size (Reber et al., 2010), to investigate this possibility, we computed the difference between averaged trial response and the correct answer (i.e., the averaged response minus the set size) for both symmetrical and asymmetrical conditions. One-sample *t*-tests on these difference values against 0 were performed in every set size, where a significantly positive difference would signify an overestimation, and vice versa. We observed no significant differences except set size 13 where an underestimation is marginally significant [set size 5: *t*(27) = 0.812, *p* = 0.424 (uncorrected); set size 6: *t*(27) = 0.273, *p* = 0.787 (uncorrected); set size 7: *t*(27) = 0.902, *p* = 0.375 (uncorrected); set size 8: *t*(27) = -0.420, *p* = 0.678 (uncorrected); set size 9: *t*(27) = 1.495, *p* = 0.146 (uncorrected); set size 12: *t*(26) = -0.702, *p* = 0.489 (uncorrected); set size 13: *t*(26) = -1.995, *p* = 0.057 (uncorrected)] (Figure 5B).

#### Exploratory Analyses: Secondary Tasks

To explore whether there is a relationship between arithmetic and visual judgment abilities with subitizing, we averaged accuracy and error distance across all set sizes from the subitizing task, and ran a correlation analysis with the secondary tasks. There was no significant correlation between the performance of the participants on our arithmetic task with subitizing accuracy [*r*(28) = 0.051, *p* = 0.797] and error distance [*r*(28) = -0.160, *p* = 0.417]. The same was true for visual symmetry judgment as well [accuracy: *r*(28) = 0.103, *p* = 0.601; error distance: *r*(28) = 0.130, *p* = 0.508].

For sensory precision, we averaged Weber fractions between set size 5 and 13 (Anobile et al., 2018) to obtain a summary precision index. We then performed a correlation analysis between precision index and z score of accuracy and RT of the participants in arithmetic and visual symmetry judgment tasks (Anobile et al., 2018). We did not observe a significant correlation between precision index and performance in the arithmetic task [z score of accuracy: *r*(28) = -0.185, *p* = 0.345; z score of reaction time: *r*(28) = 0.304, *p* = 0.116], nor the visual symmetrical task [z score of accuracy: *r*(28) = 0.138, *p* = 0.483; z score of reaction time: *r*(28) = -0.081, *p* = 0.683].

In this experiment, we have incidentally observed that answers of the participants tended to gravitate toward their responses from the previous trial. This may be similar to the previously reported “serial dependence” effect (Corbett et al., 2011; Cicchini et al., 2014; Fornaciai and Park, 2018), which we report in Supplementary Results.

### Discussion

In this experiment, we tried to get a cleaner look at the possible effect of spatial symmetry on subitizing by eliminating canonicality. We observed a positive effect of symmetry, but only at set size 6. Accuracy went from 90% in the 3–5 size range to 70% at size 6 if the shapes were asymmetrical. Yet symmetry was able to maintain accuracy of people around 90% at size 6, although subitizing performance still plummeted after that (Figure 4A). The same pattern was also observed in RT and error distance. Notably, our error distance data complemented observations from accuracy well in the sense that, even when people were incorrect in a particular trial, their incorrect responses were still closer to the right answer when the dots were symmetrical, and this facilitative effect was only significant at size 6.

When we take every trial into account and treat the participants as random factors, GLMM revealed symmetry advantage in RT. This, perhaps, provides a deeper look into the data than ANOVAs: because ANOVAs only take averaged data from each individual participant and sometimes does not include participants with missing cells (e.g., 100% accuracy), whereas GLMM takes all trials into account. This approach is, perhaps, more sensitive to the variability across trials, thus making it more difficult for the accuracy analysis to reach statistical significance while the RT effect was retained.

Although symmetry is facilitative, the fact that its effect is only confined to set size 6 suggests that the effect of symmetry is limited and weaker compared with canonicality. This is true even when we used a more liberal measure, such as the error distance. However, one can also argue that the effect of symmetry may have been masked by the elevated performance in the asymmetry control condition. That is, because each of our asymmetrical stimuli was created based off of one particular symmetrical layout (by moving 50% of the dots from the symmetrical pattern), our asymmetrical stimuli may still share too much resemblance (i.e., 50%) with the symmetrical stimuli. As such, subitizing performance may have been facilitated in the asymmetrical condition and created an unfair comparison for the effect of symmetry. Indeed, at set size 6, where the effect of symmetry is significant, the asymmetry control condition still enjoyed an accuracy of well above 70%. Therefore, to investigate whether the effect of symmetry might have been underestimated by the current design, we aim to compare the symmetrical layout with a total-random layout in Experiment 2.

## Experiment 2

**FIGURE 6:**
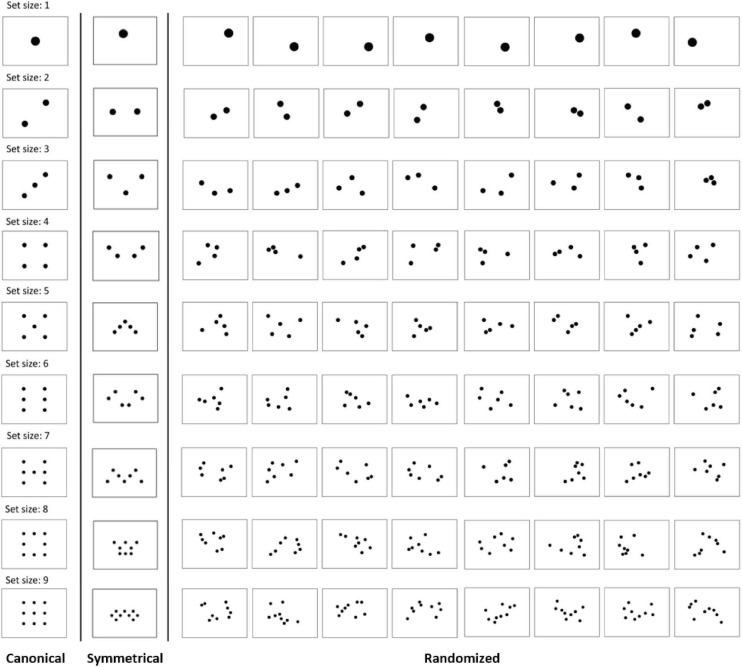
Canonical, symmetrical, and randomized designs in Experiment 2. For symmetrical figures, there were four versions for each set size (only 1 shown above, please see Figure 1 for a complete list of symmetrical designs).

In Experiment 1, we observed a facilitative effect of a symmetrical layout on subitizing at size 6. However, because our asymmetrical stimuli were created from the symmetrical stimuli (by moving 50% of the dots), these asymmetrical layouts may have been still too structural and facilitative, leading to an underestimation of the effect of symmetry. To explore this possibility, in the present experiment, we created various total-random layouts (Figure 6) to compare against symmetrical layouts. We have also included the canonical designs so that the relative facilitations between canonical and symmetrical layouts can be gauged.

### Methods

#### Participants

Thirty participants (11 males, 19 females; age, 21–37; mean age = 26.30) took part in this experiment. One participant was excluded due to abnormal vision. Other participants had normal or corrected-to-normal vision. Together, data of the remaining 29 participants were included in the data analyses. All the participants gave written informed consent prior to their participation and received financial compensation for their time. All experimental procedures were approved by the Joint Institutional Review Board of Taipei Medical University, Taiwan.

#### Subitizing Task

The trial procedure of the subitizing task was the same with Experiment 1 (Figure 1). Because canonical stimuli only consist of set sizes 1 to 9 (in most studies), we added set sizes 1 and 2 and eliminated 12 and 13 to better match with the canonical set sizes. Therefore, the set size range is from 1 to 9 in the present study. All symmetrical stimuli used here were identical as those from Experiment 1 (for a complete list, see Figure 1).

The experiment was carried out in two blocks: the canonical-vs.-randomized block, and the symmetrical-vs.-randomized blocks. In each block, the participants performed eight practice trials and 72 formal trials, resulting in a total of 80 trials. The order of the two blocks was counterbalanced between the participants.

In the canonical block, each set size had one canonical design and four randomized designs (Figure 6). The canonical design for each set size was shown four times, while the four randomized designs for each set size were shown once, resulting in 36 canonical trials and 36 randomized trials.

In the symmetrical block, set size 3 had two symmetrical designs, while other set sizes had four symmetrical designs. Each symmetrical design of set size 3 was shown two times, whereas the symmetrical designs for other set sizes were shown one time. There were also four randomized designs for each set size, and each was shown one time. Therefore, there were 36 symmetrical trials and 36 randomized trials.

#### Statistical Analyses

All accuracy and RT data were analyzed with a repeated-measures two-way ANOVA with the factors of symmetry (symmetrical vs. asymmetrical) and set size (1, 2, 3, 4, 5, 6, 7, 8, and 9). Where applicable, *post hoc* comparisons were done using Bonferroni correction. Like Experiment 1, GLMM was used for accuracy data and LMEM for RT and error distance data. Symmetrical structure and set size were modeled as fixed effects in the models. For all analyses, the ID of the participants was modeled as a random factor. All analyses were conducted using the JASP (version 0.13.1.0) software.

### Results

#### Accuracy

**FIGURE 7:**
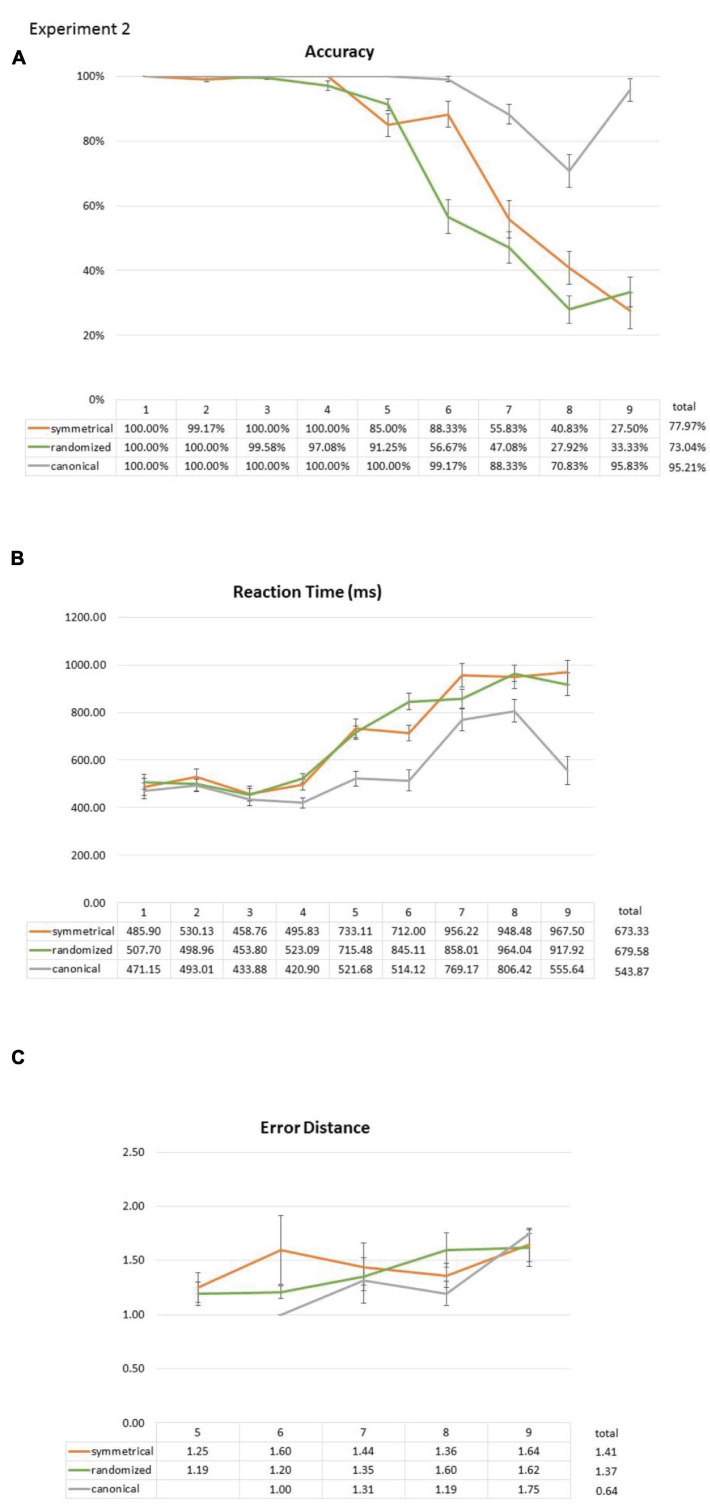
Effect of the symmetry layout on subitizing accuracy **(A)**, RT **(B)**, and error distance **(C)** compared with canonical and randomized layouts in Experiment 2. Like Experiment 1, symmetry showed advantage over the randomized layout in both accuracy and RT. Canonical layouts, on the other hand, are, by far, better than symmetrical layouts across many set sizes in both accuracy and RT. These results show that: (1) unlike canonicality, symmetry is only facilitative at set size 6, and (2) even at set size 6, the effect of symmetry is sandwiched between canonical and randomized layouts. Error bars represent standard error of the mean.

Overall, the participants had 79.81% correct trials, 3.40% no-response trials, and 16.78% incorrect trials. There were 95.21% correct trials, 2.01% no-response trials, and 2.78% incorrect trials in the canonical condition. In the symmetrical condition, there were 77.97% correct trials, 5.08% no-response trials, and 16.95% incorrect trials. In the randomized condition, there were 73.04% correct trials, 3.25% no-response trials, and 23.71% incorrect trials. A bilinear function that uses two least-squares linear slopes to fit the data across all set sizes revealed a flex point at set size 5 for the randomized layouts, 6 for the symmetrical layouts, and 7 for the canonical layouts (Figure 7A).

Accuracy data were analyzed with a repeated-measures two-way ANOVA with factors of a layout (canonical vs. symmetrical vs. randomized) and set size (1, 2, 3, 4, 5, 6, 7, 8, and 9). There was a significant main effect for both the layout [*F*(2,56) = 105.431, *p* < 0.001] and set size [*F*(8,224) = 136.062, *p* < 0.001 (Greenhouse–Geisser corrected)], as well as the interaction between them [*F*(16,448) = 27.374, *p* < 0.001 (Greenhouse–Geisser corrected)]. Subsequently, we separated all trials from both blocks into their respective symmetrical-vs.-randomized block (“symmetrical block” hereafter) and canonical-vs.-randomized block (“canonical block” hereafter), and conducted 2 × 9 two-way ANOVAs.

In the symmetrical block, two-way ANOVA showed a significant main effect for set size [*F*(8,224) = 115.217, *p* < 0.001 (Greenhouse–Geisser corrected)], a marginally significant effect for a layout [i.e., symmetrical vs. randomized; *F*(1,28) = 4.050, *p* = 0.054], and a significant interaction between set size and a layout [*F*(8,224) = 4.786, *p* = 0.002 (Greenhouse–Geisser corrected)]. *Post hoc* comparisons (all Bonferroni corrected) between symmetrical and randomized layouts in all set sizes showed significant difference only at set size of 6 [size 3: *t*(28) = 1.000, *p* = 1.000; size 4: *t*(28) = 1.440, *p* = 1.000; size 5: *t*(28) = -2.512, *p* = 0.162; size 6: *t*(28) = 4.113, *p* = 0.003; size 7: *t*(28) = 0.960, *p* = 1.000; size 8: *t*(28) = 1.890, *p* = 0.622; size 9: *t*(28) = -0.952, *p* = 1.000 (Bonferroni correction)]. This replicates our main finding from Experiment 1 (Figure 7).

In the canonical block, there was a significant main effect for both the canonical layout [i.e., canonical vs. randomized; *F*(1,28) = 142.336, *p* < 0.001] and set size [*F*(8,224) = 63.363, *p* < 0.001 (Greenhouse–Geisser corrected)], as well as the interaction between them [*F*(8,224) = 31.758, *p* < 0.001 (Greenhouse–Geisser corrected)]. *Post hoc* comparisons (all Bonferroni corrected) between canonical and randomized layouts in all set sizes showed significant differences from set sizes 5 to 9 [set size 4: *t*(28) = 1.684, *p* = 0.930; set size 5: *t*(28) = 3.550, *p* = 0.013; set size 6: *t*(28) = 6.826, *p* < 0.001; set size 7: *t*(28) = 6.620, *p* < 0.001; set size 8: *t*(28) = 5.666, *p* < 0.001; set size 9: *t*(28) = 10.369, *p* < 0.001 (Bonferroni correction)]. This implies a more robust facilitative effect in canonicality (set sizes 5 to 9) over symmetry (set size 6 only) and is consistent with previous findings, using canonical layouts (e.g., Bloechle et al., 2018).

Generalized linear mixed models with a logistic link function and a binary probability distribution for modeling single trial accuracy (i.e., correct or incorrect response) revealed a significant interaction between canonicality and set size (interaction: β = -0.113, *t* = -2.103, *p* = 0.035). Set size and a symmetrical layout also significantly affected accuracy of the participants (symmetry: β = -0.650, *t* = -2.317, *p* < 0.021; set size: β = -0.834, *t* = -13.747, *p* < 0.001). However, these numbers may have been mostly driven by the canonical layouts that showed bigger effects in the analysis above. Therefore, to better compare the effect of symmetry across Experiment 1 and 2, we, again, conducted GLMM for accuracy data only from the symmetrical block in Experiment 2. Because the symmetrical layouts from set sizes 3 to 9 are the same between both experiments, in this analysis, the only difference between Experiments 1 and 2 is the use of asymmetrical (Experiment 1) and fully randomized (Experiment 2) control stimuli. To this end, accuracy data from the symmetrical block of Experiment 2 showed that symmetry and its interaction with set size did not affect measures of accuracy (symmetry: β = -0.517, *t* = -1.618, *p* = 0.106; interaction: β = 0.048, *t* = 1.074, *p* = 0.283). Only set size significantly affected accuracy of the participants (β = -0.953, *t* = -20.181, *p* < 0.001). This is the same as what was observed from Experiment 1.

#### Reaction Time

Reaction time of all the correct trials was analyzed with repeated-measures two-way ANOVA, with the factors of the layout (canonical vs. symmetrical vs. randomized) and set size (1, 2, 3, 4, 5, 6, 7, 8, and 9). Similar to the results from accuracy, RT data also revealed a significant main effect of the layout [*F*(2,28) = 81.101, *p* < 0.001] and set size [*F*(8,112) = 44.338, *p* < 0.001 (Greenhouse–Geisser corrected)], and a significant interaction between them [*F*(16,224) = 12.641, *p* < 0.001 (Greenhouse–Geisser corrected)].

Two-way ANOVA from the symmetrical block showed a significant main effect of set size [*F*(8,56) = 36.387, *p* < 0.001 (Greenhouse–Geisser corrected)], no effect of the layout [*F*(1,7) = 1.790, *p* = 0.223], but a significant interaction between the set size and the layout [*F*(8,56) = 4.121, *p* = 0.026 (Greenhouse–Geisser corrected)]. *Post hoc* comparisons between symmetrical and randomized layouts (all Bonferroni corrected) in all set sizes showed significant difference only at set size 6 [size 1: *t*(28) = -2.863, *p* = 0.070; size 2: *t*(28) = 0.860, *p* = 1.000; size 3: *t*(28) = -0.505, *p* = 1.000; size 4: *t*(28) = -2.522, *p* = 0.158; size 5: *t*(28) = 0.740, *p* = 1.000; size 6: *t*(26) = -5.743, *p* < 0.001; size 7: *t*(24) = 2.228, *p* = 0.320; size 8: *t*(15) = -0.228, *p* = 1.000; size 9: *t*(11) = 2.553, *p* = 0.241 (Bonferroni correction)].

In the canonical block, the main effect of the set size [*F*(8,64) = 30.324, *p* < 0.001 (Greenhouse–Geisser corrected)], the layout [*F*(1,8) = 68.201, *p* < 0.001], and the interaction between them [*F*(8,64) = 14.964, *p* < 0.001 (Greenhouse–Geisser corrected)] was all statistically significant. *Post hoc* comparisons (all Bonferroni corrected) between canonical and randomized layouts in all set sizes showed significant differences at set sizes 1, 4, 5, 6, 8, and 9 [size 1: *t*(28) = -3.958, *p* = 0.004; size 2: *t*(28) = 0.532, *p* = 1.000; size 3: *t*(28) = -1.396, *p* = 1.000; size 4: *t*(28) = -4.849, *p* = 0.004; size 5: *t*(28) = -9.573, *p* < 0.001; size 6: *t*(21) = -6.873, *p* < 0.001; size 7: *t*(21) = -2.861, *p* = 0.084; size 8: *t*(16) = -4.419, *p* = 0.004; size 9: *t*(18) = -7.839, *p* < 0.001 (Bonferroni correction)].

Linear mixed models showed a significant interaction between the canonical layout and the set size (β = 18.306, *t* = 12.682, *p* < 0.001), whereas the interaction between the symmetrical layout and the set size was only marginally significant (β = -2.991, *t* = -1.827, *p* = 0.068). Both the set size and the canonical layout significantly affected the RT of the participants (canonical structure: β = -18.676, *t* = -2.523, *p* = 0.012; set size: β = 51.796, *t* = 30.413, *p* < 0.001). If we exclude canonical layouts and focus on the symmetrical block alone, both symmetry and set size can significantly predict the RT of the participants (set size: β = 70.083, *t* = 36.125, *p* < 0.001; symmetry: β = 20.590, *t* = 2.255, *p* = 0.024), and their interaction was marginally significant as well (β = -3.386, *t* = -1.754, *p* = 0.079). The positive coefficient of symmetry in GLMM of RTs indicates significantly faster RT in symmetrical trials over randomized trials. Predicted RT of the Participants was equal to 337.572 + 70.083 ^∗^ set size + 20.590 ^∗^ symmetry -3.386 ^∗^ set size ^∗^ symmetry. These findings are also consistent with our observations from Experiment 1.

Lastly, bilinear fitting showed a flex point at set size 4 for the randomized layouts, also 4 for the symmetrical layouts, and 6 for the canonical layouts (Figure 7B), which is quite consistent with the RT results from earlier studies (e.g., Mandler and Shebo, 1982).

#### Error Distance

Error distance was computed for every incorrect trial. However, the number of incorrect trials in canonical condition was too low (2.78% of total canonical trials); thus, we excluded the canonical condition from the following analysis. Two-way ANOVA with factors of the layout (symmetrical vs. randomized) and the set sizes (6, 7, 8, and 9) did not show any significant main effect of interaction [the symmetrical layout: *F*(1,4) = 2.949, *p* = 0.161; set size: *F*(3,12) = 0.803, *p* = 0.466 (Greenhouse–Geisser corrected); interaction: *F*(3,12) = 0.927, *p* = 0.420 (Greenhouse–Geisser corrected)]. Using LMEM, including all incorrect trials (19.73% of total trials) from the symmetrical block, the analysis showed that set size significantly affected error distance of the participants (β = 0.161, *t* = 3.822, *p* < 0.001). However, symmetry and the interaction between set size and symmetry were not significant (symmetry: β = -0.298, *t* = -0.914, *p* = 0.358; interaction: β = 0.041, *t* = 0.986, *p* = 0.322).

#### Weber Fraction

**FIGURE 8:**
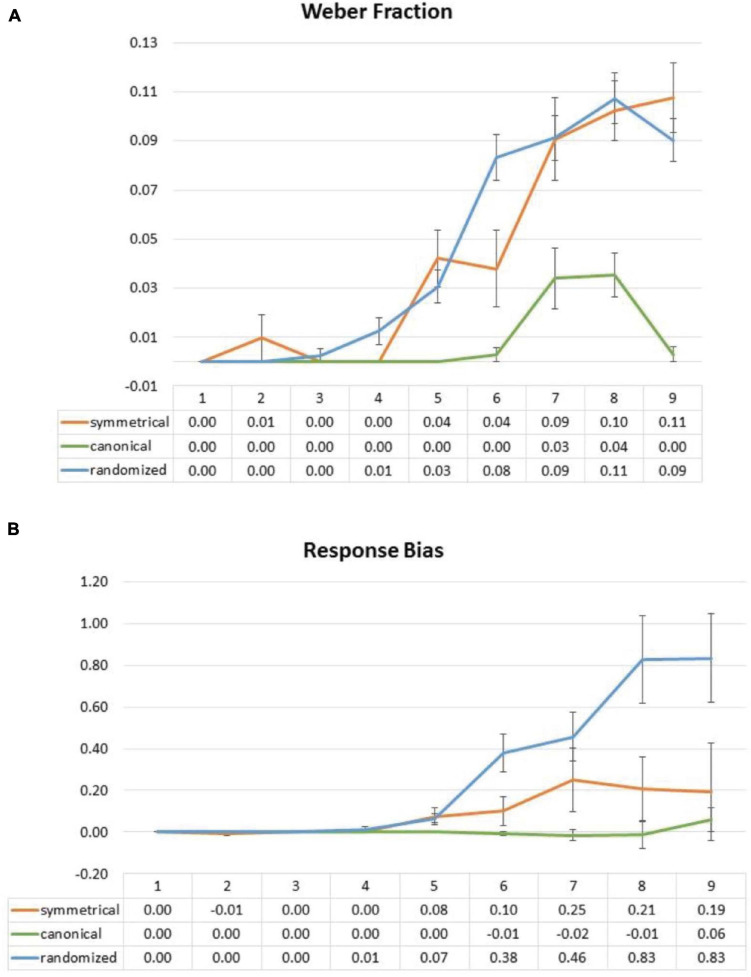
Effect of symmetrical structure on Weber fraction **(A)** and response bias **(B)** in Experiment 2. Weber fraction data showed no significant interaction between the conditions. For response bias, there was no significance responses biases between canonical and symmetrical, but the randomized layout led to significant overestimation biases from set sizes 5 to 9.

Response standard deviation for each set size and condition of each participant was divided by the corresponding average perceived numerosity. These Weber fraction ratios were submitted to a two-way ANOVA with factors of the layout (symmetrical vs. canonical vs. randomized) and set sizes (1 to 9). There was a significant main effect of layout [*F*(2,58) = 0.100.945, *p* < 0.001] and a significant interaction between the layout and the set size [*F*(16,464) = 13.601, *p* < 0.001]. However, in the symmetrical block, a two-way ANOVA with factors of the layout (symmetrical vs. randomized) and the set sizes (1 to 9) did not show any significant main effect of the layout and interaction [layout: *F*(1,29) = 0.103, *p* = 0.751; interaction: *F*(8,232) = 1.628, *p* = 0.171 (Greenhouse–Geisser corrected]. It seems that sensory precision of the symmetrical layout was not significantly different from the randomized layout (Figure 8A).

#### Response Bias

In order to investigate whether the symmetrical structure may bias the numerosity estimation in Experiment 2, we, again, contrasted responses of the participants with set size and submitted these contrasts to a one-sample *t*-test against 0 (Figure 8B). We observed no difference from 0 in every set size [set size 5: *t*(28) = 1.797, *p* = 0.083 (uncorrected); set size 6: *t*(28) = 1.485, *p* = 0.149 (uncorrected); set size 7: *t*(28) = 1.542, *p* = 0.134 (uncorrected); set size 8: *t*(28) = 1.292 *p* = 0.207 (uncorrected); set size 9: *t*(28) = 0.652, *p* = 0.520 (uncorrected)]. However, and surprisingly, randomized layouts produced significant overestimation biases across most set sizes [set size 5: *t*(29) = 3.376, *p* = 0.002 (uncorrected); set size 6: *t*(29) = 4.111, *p* < 0.001 (uncorrected); set size 7: *t*(29) = 4.024, *p* < 0.001 (uncorrected); set size 8: *t*(29) = 3.722, *p* < 0.001 (uncorrected); set size 9: *t*(29) = 3.719, *p* < 0.001 (uncorrected)], which may be a complementary effect that mirrors the underestimating bias effect in symmetrical stimuli (hence making the randomized layout appear larger), although there is not enough evidence in our data to confirm either way.

### Discussion

In this experiment, symmetrical layouts from Experiment 1 were used to compare with canonical and randomized layouts. There are two observations worth noting here: (1) for symmetrical layouts, we were able to replicate the same advantageous effect that occurs only at set size 6, and (2) canonical layouts had a bigger facilitative effect than symmetrical layouts.

First, the randomized condition here had lower accuracy (<60%) at set size 6 than the asymmetrical condition from Experiment 1 (>70%), which confirms the initial suspicion that the 50%-randomized asymmetrical condition from Experiment 1 was not random enough and thus created an unfair comparison for the symmetrical effect. However, despite the full randomization manipulation here, we still did not observe a greater facilitative effect for the symmetrical layouts. Instead, the set size 6 effect is still observed here with a lower baseline control to compare against. Therefore, we think that symmetry really is only facilitative at set size 6 where people are at the brink of their processing capacity.

Second, canonical layouts were able to produce greater facilitation to subitizing performance, both over symmetrical and randomized layouts, and across multiple set sizes even beyond 6 (Figure 7). Therefore, it is quite clear that symmetry is not the same as canonicality. Moreover, as such, previous findings on canonicality and subitizing cannot be explained by spatial symmetry alone.

Lastly, trial-to-trial priming effect from Experiment 1 is no longer observed here in Experiment 2. Given that the only change between the two experiments is the amount of randomization, we can only assume that the 50%-randomized asymmetrical layouts from Experiment 1 were structured enough that prompted the participants to employ some kind of template-matching strategy. Otherwise, the amounts of a perceptual load between the experiments are the same, and thus elimination of the priming effect is best attributed to the response task set of the participants. We discuss this in more detail in General Discussion.

### General Discussion

The purpose of this study is to investigate the possible effect of spatial symmetry on subitizing by using symmetrical but non-canonical shape structures. Previous studies mostly focused on the facilitative effect of canonical structures, yet, coincidentally, all canonical structures happened to be symmetrical (except sizes 2 and 3). Therefore, it is important to single out symmetry alone and examine its effect independently from canonicality.

In Experiment 1, symmetrical vs. 50%-randomized asymmetrical comparison showed an advantageous effect of symmetry at set size of 6. People were about 90% accurate in the 3–5 size range regardless of symmetry, but this number plummeted to 70% at size 6 if the shapes were asymmetrical. Symmetry was able to maintain accuracy of people around 90% at set size 6. Beyond that, there was no facilitative effect of symmetry at set size 7 and beyond (Figure 4A). This effect at set size 6 is observed again in Experiment 2 when symmetry is being compared against 100%-randomized condition.

#### Is Canonicality the Same as Symmetry?

Can the present observations from this study explain previous findings on canonical shapes? Based on our results from Experiment 2, we think the answer is a clear “no”: Canonical layouts were able to produce greater advantages in both subitizing accuracy and RT, and across multiple set sizes beyond set size of 6 (Figure 7). Even from Experiment 1, the absence of an improvement at set sizes 3 and 4 suggests that symmetry was not robust enough to show an effect in ceiling conditions. Yet, at set size of 7, where things get too difficult, symmetry also could not help the participants to subitize more accurately, not even when we used a more liberal measure like error distance (Figure 4C). Therefore, the fact that the facilitative effect of symmetry is confined to only set size of 6 seems to suggest that the effect is weaker and only protect accuracies of people in the borderline capacity condition.

To put our results in the context of previous findings, canonical shapes of Wender and Rothkegel (2000) facilitated subitizing performance even at set size 4, where people were already performing at a ceiling level. Similarly, Krajcsi et al. (2013) also found that both canonical and jittered conditions were much faster than the total-random condition even at set size of 4. Finally, Bloechle et al. (2018) found RT facilitation at set size 5 and Piazza et al. (2002) at set size 6. Meanwhile, our symmetry facilitation in RT was marginally significant and not as robust as the previous studies found.

It is important to note that findings from these studies mentioned above are quite remarkable, as it is much more difficult to improve performance of one at smaller set sizes because subitizing in this range is automatic (Mandler and Shebo, 1982). As such, it would take a much more robust effect to facilitate an already-automatic subitizing process at sizes 3 to 5. Therefore, although one can argue that previous findings on canonicality have been a combination of canonicality *and* symmetry, our results suggest that it is safe to say that most of their observed effects should be attributed to canonicality and not symmetry, because the amount of facilitation from symmetry alone is insufficient to show significant speeding effect over randomized layouts in set sizes of 5 and below.

#### Perceptual Grouping and Pattern Recognition

The weaker effect of symmetry possibly suggests a different process from shape canonicality. We speculate that the effect of symmetry may be part of the perceptual grouping mechanism, recently known as “groupitizing” (Starkey and McCandliss, 2014), which can be different from the pattern recognition processes of canonical structure (Mandler and Shebo, 1982). Several recent studies have documented that the Gestalt-like grouping principles, such as spatial proximity and stimuli similarity, can all lead to an advantage in numerical subitizing and estimation (Anobile et al., 2020; Ciccione and Dehaene, 2020; Moscoso et al., 2020). In particular, Ciccione and Dehaene (2020) showed their participants multiple dots that were separated into several groups of same or different shapes (e.g., nine dots grouped into three sets of same/different triangular organizations) and observed strongest facilitation in the “same number and same shape” condition. That is, the participants could perform mental multiplication, such as 3 × 3, when they were able to break down bigger set sizes *via* perception grouping. Incidentally, one study by Delvenne et al. (2011) found a similarly weak facilitative effect in subitizing by using grouping manipulations in both visual fields. Therefore, it may be possible that perceptual grouping mechanisms can be weakly facilitative around set sizes of 5 or 6 where it is easier to push subitizing performance over the border. One event-related potentials study by Mazza and Caramazza (2012) showed that earlier components, such as N2pc, can be sensitive to object grouping and individuation during the subitizing process, but not later components, such as the contralateral delayed activity. In this light, we conjecture that there is no difference between symmetry and groupitizing; namely, the participants could simply recognize half of the figure and multiply it by 2 such that symmetry can be viewed as a subset example of the general groupitizing process.

Is groupitizing the only mechanism at play here? Our RT data, along with the bilinear analysis from Experiment 2 (Figure 7B), do show two visible slopes from 1 to 4 and from 4 and on, which is consistent with previous reports, using canonical designs (Mandler and Shebo, 1982; Krajcsi et al., 2013; Bloechle et al., 2018). However, note that the flex point for the canonical condition is visibly different from those from the symmetrical (and the randomized) conditions (Figure 7B). Yet, from a groupitizing point of view, the groupitizing advantage is presumably equal between the symmetrical and canonical conditions because both patterns afford mental multiplication of 2. Therefore, we speculate that the stronger facilitation offered by canonical organization over symmetry is better explained by schema-like pattern-recognition facilitation. This is based on the fact that any patterns can be learned to become canonical (Lassaline and Logan, 1993), which implies a rapid memory access mechanism, and can possibly be linked to the automatic processing of “gist” that takes as short as only 50 min to complete (Sampanes et al., 2008). As previously mentioned, this may also be similar to the memory retrieval effect in experts, where a brief exposure of the chessboard is enough to activate long-term memory representations of the semantics. Here, the strongest form of pattern recognition account would predict a flat RT slope across all set sizes, because recognizing a well-remembered three-dot scene or nine-dot scene should not be any different. Although this prediction sounds quite bold, this is, indeed, true in our RT data (Figure 8B) if we ignore set sizes 7 and 8, which are the less-used canonical patterns in everyday life. Specifically, notice how RT between set size 6 (514.12 ms) and 9 (555.64 ms) in the canonical condition is almost identical despite a 33% difference in dots. In contrast, the RT from the symmetrical condition shows a more linear increase from 712 ms (set size 6) to 967.5 ms (set size 9). Indeed, if not for the familiar configuration that is already encoded in long-term memory system of one, any cognitive processing or judgment processes about unfamiliar (but symmetrical) configurations would, at least, take some time to process, which would likely to result in non-significant effects in the low set size, ceiling conditions. Therefore, in the context of this study, we think our symmetrical effect may have been mostly driven by groupitizing, and canonical effect by visual pattern recognition. These (at least) two sources, or layers, of facilitation here also highlight the fact that subitizing is, perhaps, a multifaceted process, a point that has been raised by many recently (e.g., Katzin et al., 2019).

#### Conclusion

Taking both Experiments 1 and 2 together, our results showed that symmetry facilitates subitizing performance, but only at set size of 6, which suggests that the effect is insufficient to improve performance of people in the lower or upper range. Comparing with previous studies on the effect of canonical shapes on subitizing, we conclude that, although previous findings mixed symmetry in their canonical shapes, their findings on shape canonicality cannot be explained by symmetry alone. Our results also suggest that groupitizing and pattern recognition mechanisms are not mutually exclusive and may both be at play here for subitizing.

## Data Availability Statement

The raw data supporting the conclusions of this article will be made available by the authors, without undue reservation.

## Ethics Statement

The studies involving human participants were reviewed and approved by the Joint Institutional Review Board, Taipei Medical University. The patients/participants provided their written informed consent to participate in this study.

## Author Contributions

C-YH designed the experiment, collected data, analyzed data, and wrote the manuscript. Y-HL analyzed data and wrote the manuscript. PT designed the experiment, analyzed the data, and wrote the manuscript. All authors contributed to the article and approved the submitted version.

## Conflict of Interest

The authors declare that the research was conducted in the absence of any commercial or financial relationships that could be construed as a potential conflict of interest.

## Publisher’s Note

All claims expressed in this article are solely those of the authors and do not necessarily represent those of their affiliated organizations, or those of the publisher, the editors and the reviewers. Any product that may be evaluated in this article, or claim that may be made by its manufacturer, is not guaranteed or endorsed by the publisher.

## Footnotes

1 We thank the reviewer for this suggestion.
