# Supplementary material for: Effect of Non-canonical Spatial Symmetry on Subitizing
Source: Front Psychol. 2021 Jul 29;12:562762. doi: 10.3389/fpsyg.2021.562762 (PMC8358310; doi:10.3389/fpsyg.2021.562762)
Supplement: Supplementary file 1 [file Data_Sheet_1.PDF]

Supplementary Results

In Experiment 1, we noticed that participants' answers tended to gravitate toward their responses from the previous trial. This is been previously reported as the "serial dependence" effect (Cicchini et al., 2014; Corbett et al., 2011; Fornaciai & Park, 2018). Although this was not originally planned in the current study, we conducted the following exploratory analysis based on such observation and to further explore the mistakes that the participants made. We plotted participants' error distance (overestimation vs. underestimation) against the *numerical* distance between the last trial and the current trial. For example, if participants saw 9 discs in the previous trial and saw 4 discs in the current trial, the actual between-trial numerical distance is a positive 5. This plot would give us some insight to the influence of previous trial, if any. As shown in Supplementary Figure 1, there is a very strong linear trend in the positive direction, meaning that when participants get the current trial wrong, the direction and magnitude of their error distance is somewhat proportional to the set size from the previous trial, as if their responses for the current trial is gravitating towards the numerosity of previous trial. A simple linear regression was calculated to predict the error distance based on numerical distance between the last trial and the current trial. A significant regression equation was found [F(1,360) = 27.761, p <0.001], with an R^2 of 0.072. Participants' predicted error distance was equal to 0.290 + 0.115 * numerical distance. Participants' average error distance increased 0.115 for each numerical distance increment. LMEM with both slopes and participant IDs as random factors also showed significant effect of numerical distance ($\beta = 0.116$, t = 3.818, $p < 0.001$), where participants' predicted error distance was equal to 0.348+0.116*numerical distance.

Based on this relationship between the set size of the previous trial and the error distance of the current trial, we included set size of previous trial as a covariate in regression models for accuracy, and reaction time. Models with and without previous trial information are then compared using chi square statistics to compare how well they fit the data. For accuracy, only set size was significant in GLMM with fixed effect factors as set size and symmetry. Even though including previous item as a covariate into the GLMM, no significant difference was observed in these two model (model 1: glmer(ACC ~ set size + symmetry + prior + set size : symmetry + (1|participant), family = binomial); model 2: glmer(ACC ~ set size + symmetry + set size : symmetry + (1|participant), family = binomial); $X^2(1) = 0.663$, $p = 0.416$). In terms of RT, the model with previous trials provided a better fit to the RT data (model 1: lmer(RT ~ set size + symmetry + prior+ set size : symmetry + (1|participant)); model 2: lmer(RT ~ set size + symmetry + set size : symmetry + (1|participant)); $X^2(1) = 9.839$, $p = 0.002$). In regression analysis predicting RT (of correct trials) from the set size of previous trial,

set size, symmetrical structure was equal to 389.307+78.319*set size+55.301*symmetry+6.508*prior trial-8.561*set size*symmetry.

In Experiment 2, we aimed to conduct the same analysis as above and conducted a simple linear regression to investigate participants' error distance (overestimation vs. underestimation) against the *numerical* distance between the last trial and the current trial. However, unlike Experiment 1, linear regression did not show any significant effect. LMEM with both slopes and participant IDs as random factors also showed no significant effect of numerical distance ($\beta = 0.027$, $t = 1.258$, $p = 0.214$).

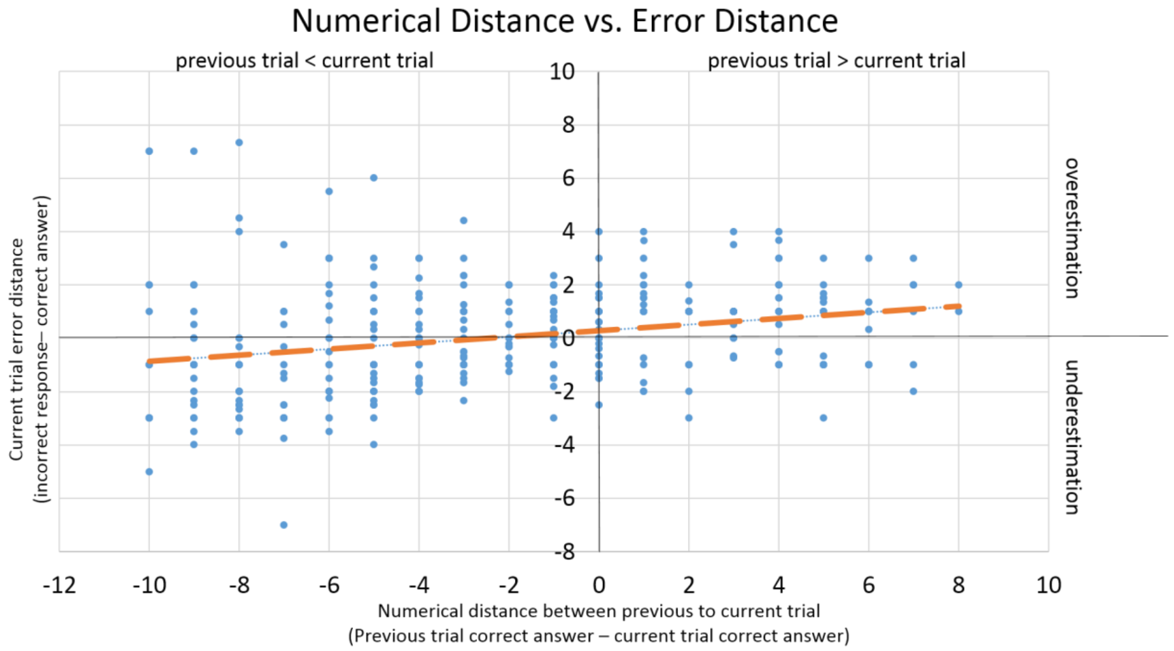

Supplementary Figure 1: Trial-to-trial priming, or serial dependence effect, from Experiment 1. The numerical difference between previous trial and current trial is marked on X-axis, and participants' error distance is marked on Y-axis. Here it is shown that when participants saw a bigger set size in the immediately preceding trial, they also tend to make overestimations in the current trial. This overestimation also increases in magnitude as the trial-to-trial difference increases, showing a positive regression line. The same is true for underestimations, where preceding trials with smaller set size also leads to underestimations in the current trial in a linear way.
